# Inflammatory Bowel Diseases and Sarcopenia: The Role of Inflammation and Gut Microbiota in the Development of Muscle Failure

**DOI:** 10.3389/fimmu.2021.694217

**Published:** 2021-07-13

**Authors:** Olga Maria Nardone, Roberto de Sire, Valentina Petito, Anna Testa, Guido Villani, Franco Scaldaferri, Fabiana Castiglione

**Affiliations:** ^1^ Gastroenterology, Department of Clinical Medicine and Surgery, University Federico II of Naples, Naples, Italy; ^2^ Department of Medicine and Translational Surgery, Fondazione Policlinico Universitario “A. Gemelli” IRCCS, University Cattolica del Sacro Cuore, Rome, Italy

**Keywords:** IBD, sarcopenia, gut-muscle axis, gut microbiota, probiotics, inflammation, muscle wasting, malnutrition

## Abstract

Sarcopenia represents a major health burden in industrialized country by reducing substantially the quality of life. Indeed, it is characterized by a progressive and generalized loss of muscle mass and function, leading to an increased risk of adverse outcomes and hospitalizations. Several factors are involved in the pathogenesis of sarcopenia, such as aging, inflammation, mitochondrial dysfunction, and insulin resistance. Recently, it has been reported that more than one third of inflammatory bowel disease (IBD) patients suffered from sarcopenia. Notably, the role of gut microbiota (GM) in developing muscle failure in IBD patient is a matter of increasing interest. It has been hypothesized that gut dysbiosis, that typically characterizes IBD, might alter the immune response and host metabolism, promoting a low-grade inflammation status able to up-regulate several molecular pathways related to sarcopenia. Therefore, we aim to describe the basis of IBD-related sarcopenia and provide the rationale for new potential therapeutic targets that may regulate the gut-muscle axis in IBD patients.

## Introduction

The European Working Group on Sarcopenia in Older People defined sarcopenia as a progressive and generalized skeletal muscle disorder, characterized by loss of muscle mass and function, low muscle strength and poor physical performance (1). Accordingly, it represents a major health burden in industrialized country by determining the risk of physical disability, poor quality of life, increased hospital admissions and increased mortality (2, 3).

Muscle impairment represents a common pathological hallmark of common chronic gastrointestinal diseases, including inflammatory bowel diseases (IBD). Recently, it has been reported that 42% of IBD patients suffered from sarcopenia (4). In addition, in most of them sarcopenia coexists with malnutrition as results of chronic inflammation.

Inflammatory bowel disease, including Crohn’s disease (CD) and ulcerative colitis (UC), are chronic inflammatory disorders affecting the gastrointestinal tract, characterized by a relapsing-remitting course. Although their etiopathogenesis is still unknown, it has been hypothesized an aberrant immune-mediated response to specific antigens of the gut microbiota (GM) in genetically predisposed individuals (5–9).

The GM represents a real ecosystem, consisted of more than 10^14^ bacteria and more than 1000 species as well as fungi, viruses, phages, parasites, and archea, that colonizes gastrointestinal tract and plays an important role in nutrient absorption, maintenance of metabolic homeostasis, protection from infections and development of systemic and mucosal immunity (10–13).

Several studies have shown significant difference in the GM composition between patients with IBD and healthy people. In particular, the phylum Firmicutes - specifically Faecalibacterium prausnitzii - is often reduced in the stool of patients with CD, while members of the Proteobacteria phylum, such as Enterobacteriaceae, including Escherichia coli, are commonly increased in patients with IBD compared to healthy individuals (5, 14–16). This contributes to a shift in the balance between commensal and potentially pathogenic microorganism that leads to dysbiosis (16).

Among several factors involved in the pathogenesis of sarcopenia, the role of GM in developing muscle wasting in IBD patients has now gained increasing interest. It has been hypothesized that GM moving from protective to pro-inflammatory effects, might alter the immune response and host metabolism, promoting a low-grade inflammation status able to up-regulate several molecular pathways related to sarcopenia, with consequent development of musculoskeletal impairment and frailty (17–19).

Therefore, this narrative review aims to describe the bases of IBD-related sarcopenia and to provide the rationale for new potential therapeutic targets that might regulate the gut-muscle axis in IBD patients.

## The Gut-Muscle Axis Hypothesis

**Figure 1:**
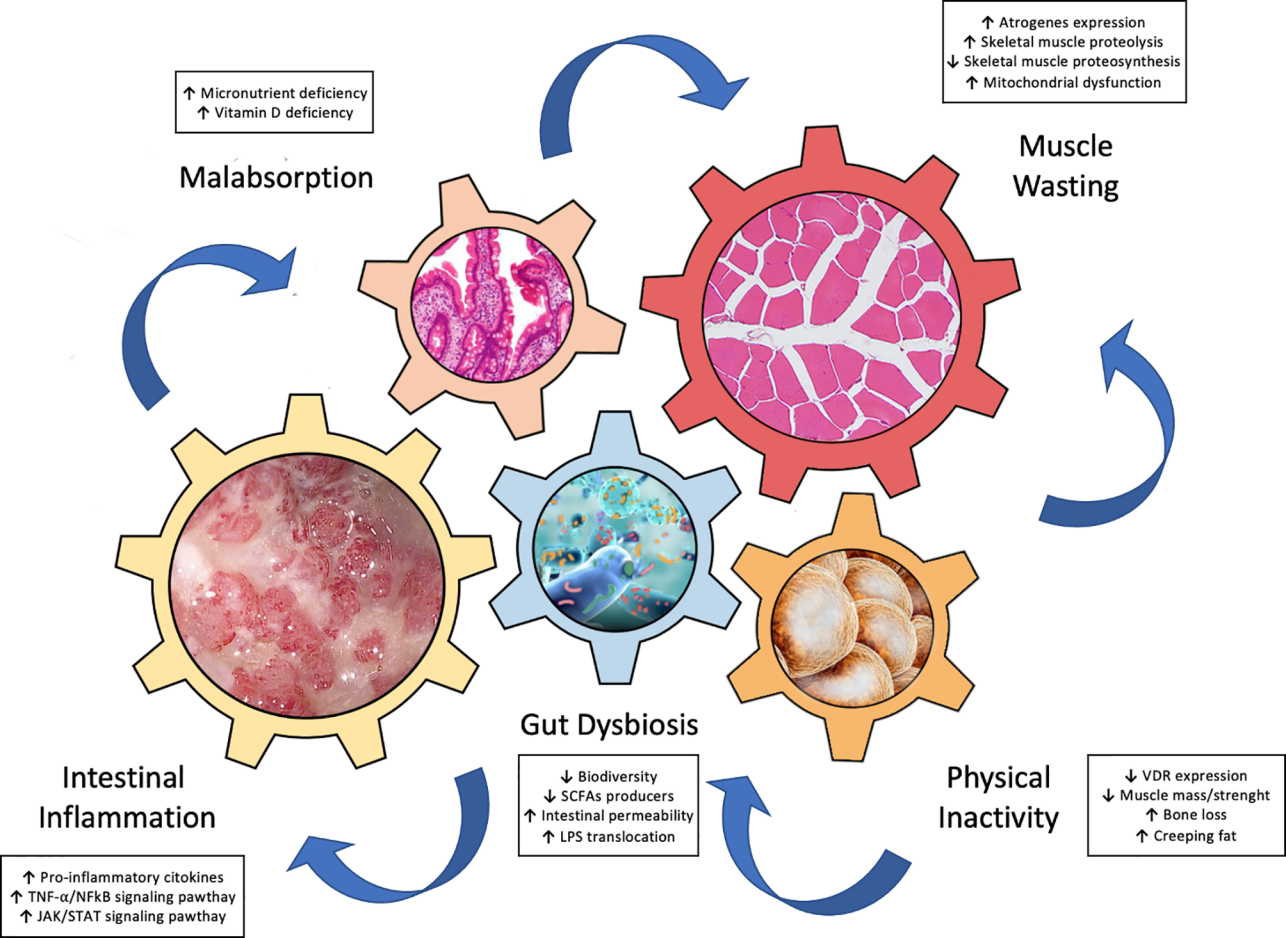
Key drivers involved in the pathogenesis of “gut-muscle axis”.

Recently, growing data support the hypothesis of a “gut-muscle axis” (5, 20, 21), wherein inflammation, gut dysbiosis, and malnutrition, interplay chorally for development of muscle failure in IBD patients (**Figure 1**). In this next section we focus on these key players of the gut-muscle axis.

### Inflammation

The reduction of muscle mass and strength in sarcopenia increases with age. There are several factors involved in the development of muscle atrophy and age-related sarcopenia. The persistent low-grade inflammatory status in the elderly, characterized by increased circulating levels of pro-inflammatory cytokines, such as TNF-alpha, IL-6, and myostatin, defined as “inflammaging”, is crucial (22–24). To date there were conflicting data with regards to the median ages of sarcopenic IBD patients. Zhang et al. reported that IBD patients with sarcopenia were significantly younger compared with those without sarcopenian (6), while according to Pedersen et al. sarcopenic patients were more likely to be older with more medical comorbidities, such as hypertension and diabetes, than younger non sarcopenic (25).

However, the intestinal inflammatory state that characterizes patients with IBD might be considered as the starting point for the development of muscle impoverishment, by activating several pathways in common with sarcopenia (5, 17, 19).

TNF-alpha, produced by macrophages, lymphocytes, mast cells, fibroblasts and endothelial cells, is considered the key driver of intestinal damage, by stimulating macrophages to produce pro-inflammatory cytokines, inducing apoptosis of intestinal epithelial cells and Paneth cells, and stimulating the synthesis of proteases (26, 27). It is commonly believed that the disruption of intestinal epithelial tight junctions (TJ) leads to an increase of gut permeability with a consequent translocation of lipopolysaccharide (LPS) into systemic circulation. Several studies showed that the epithelial barrier function is impaired in IBD patients. Experimental models of colon biopsies of IBD patients hypothesized as a possible cause of barrier dysfunction a reduction of tight junction strands in both UC and CD (28–31). In response to the LPS stimuli, nuclear factor κB (NF-kB) translocates from cytoplasm to nucleus and stimulates dendritic cells and macrophages to produce pro-inflammatory cytokines and mediators, for instance, cyclo-oxygenase-2 (COX-2), TNF-alpha, inducible nitric oxide synthase (iNOS), and IL-6, that regulate intestinal and systemic inflammation (28–31).

Importantly, it has been shown that increased serum levels of TNF-alpha are associated with muscle impairment. Indeed, TNF-alpha regulates the activation of NF-kB signaling pathway through the expression of the “atrogenes” (atrophy-related genes) and promotes protein degradation through the transcription of ubiquitin proteasome E3 ligases: muscle RING-finger protein-1 (MurF1), and Atrogin (32, 33).

In addition, IL-6 produced by macrophages and T cells in the inflamed gut, is a pleiotropic cytokine able to upregulate the production of pro-inflammatory cytokines and inhibit T cell apoptosis through the activator of transcription 3 (STAT3) and hence contributes to the disruption of skeletal muscle proteosynthesis (34, 35).

Notably, the role of IL-6 in muscle homeostasis depends on the timing of its production. While during exercise IL-6 transient production is associated with beneficial effects, a persistent elevation of its serum levels, in particular in elderly, is associated with muscle wasting and sarcopenia (36, 37). Of note, the reduced proteosynthesis and the increase in protein degradation of the skeletal muscle tissue seem to be promoted by the activation of three different cellular signaling pathways, all starting from the binding of IL-6 and its receptor: the Janus kinasi (JAK)/STAT pathway, the Mitogen-activated protein kinase (MAPK)/Extracellular signal-regulated kinases (ERK) pathway and the Phosphoinositide 3-kinase (PI3K)/Protein kinase B (AKT)/mammalian target of rapamycin (mTOR) pathway (38).

In particular, the JAK/STAT signaling pathway is highly involved in IBD pathogenesis, mediating the function of several inflammatory cytokines implicated in gut inflammation, such as IL-2, IL-4, IL-6, IL-7, IL-9, IL-12, IL-15, IL-21, IL-23, and IFN-gamma (39).

In this context, skeletal muscle fibers by expressing cytokine receptors and both the toll-like receptor (TLR)-2 and TLR-4 (which bind LPS), determine an overproduction of reactive oxygen species (ROS) and an oxidative stress (40). Given that mitochondria are the most vulnerable cellular organelle to ROS, the oxygen consumption decreases mitochondrial respiration and the ability to produce ATP. This process drives to disrupted mitochondrial dynamics and leads to “mitophagy”, i.e. hyperactivation of mitochondrial degradation pathways (41).

In a number of chronic clinical disorders, such as also sepsis, heart failure and chronic obstructive pulmonary disease, mitochondrial dysfunction has been associated with increased systemic inflammation, which influences muscle protein synthesis and impairs both mitochondrial and muscle function (42). These processes are predominantly a consequence of oxidative stress secondary to ROS with a negative impact on skeletal muscle and hence are likely to contribute to the development of sarcopenia (42).

Although mitochondrial dysfunction has been founded in the intestinal epithelium of IBD patients, the role of epithelial mitochondrial stress in the pathogenesis of IBD has not yet fully understood (43). A recent study conducted by Jackson et al. identify Paneth cells as highly susceptible to mitochondrial dysfunction driven by loss of prohibitin 1 (PHB1), a major component protein of the inner mitochondrial membrane, and central to the pathogenesis of ileitis (44). This provides important translational implications for mitochondrial-targeted therapeutics in a subset of CD patients exhibiting Paneth cell defects (44).

Finally, there is a strict connection between mitochondria function and microbiota. In fact, the short chain fatty acids (SCFA) produced by gut bacteria is positively correlated with the expression of mitochondrial protein involved in the energy production, redox balance, and the modulation of the inflammatory cascade activation. However, in IBD patients, bacteria that ferment fibers and produce SCFAs are typically reduced in mucosa and feces as compared to healthy individuals. This low representation of SCFA producers in gut microbiota has been associated with increased subclinical chronic inflammation, which reinforces the skeletal muscle anabolic resistance (45).

### Gut Dysbiosis

Gastrointestinal tract and skeletal muscle tissue interact each other through a complex network modulated by the GM consisting of hormones, implicated in the homeostasis of energy metabolism, protein metabolism favors breakdown, and inflammatory mediators (i.e.TNFα) which increase the permeability of the intestinal membrane and cause both local and systemic inflammatory effects (18, 20, 21).

In IBD patients, this ecosystem is altered in terms of biodiversity, microbial composition and functions, determining the so-called “gut dysbiosis”, that reflects an inappropriate immune response of a complex microbial community to the intestinal inflammation (46–48). It has been reported that IBD patients had a decrease of Firmicutes and Bacteroides, and a relative increase of bacterial species belonging to Enterobacteriaceae, that disrupt the intestinal barrier integrity, and an increase in mucolytic bacteria that determine the degradation of the mucosal barrier and thereby an increased penetration of pathogens into the intestinal tissues (49). This condition, defined as “leaky gut syndrome”, represents a cofactor in the onset of a pro-inflammatory status leading to sarcopenia in both IBD and elderly patients (50, 51). Several studies showed an overall decrease in alpha and beta diversity of the gut microflora in IBD patients (52, 53). It has been reported by Qin et al. that in IBD patients’ mucosal microbial genes are reduced of 25% compared to healthy controls (54). Recently, a comprehensive description of qualitative alterations in the gut microbiome of 132 patients affected by IBD has shown the presence of a functional dysbiosis in the gut microbiome during flairs of the disease with an increase in facultative anaerobes over obligate anaerobes, as well as molecular dysregulation in microbial transcription, metabolites, and serum levels of antibodies in the host (55). Particularly, Joossens et al. reported an increase in Ruminococcus gnavus and a decrease in Bifidobacterium adolescentis, Dialister invisus, Faecalibacterium prausnitzii, and an unknown member of Clostridium cluster XIVa in IBD patients (56).

However, GM influences resident mucosal immune cells to produce proinflammatory cytokines and microbiota‐derived metabolites. Among them, SCFAs, secondary bile acids, water‐soluble B‐vitamins, tryptophan, polyphenols, and urolithins, are crucial for modulation of gut-muscle axis, by promoting insulin sensitivity, biosynthesis of amino acids, mitochondrial biogenesis, and muscular anabolism (21, 57–61).

Of note, several studies investigated the modulatory role of GM on the skeletal muscle functions and amino acid bioavailability (62–64). For instance, in both frail and IBD population there is a decrease in Faecalibacterium prausnitzii, a SCFAs producer with a significant anti-inflammatory function. In addition, Bifidobacteria lactobacilli, involved in protein breakdown to amino acids within the gut, produce SCFAs for energy production, stimulate insulin growth factor -1 (IGF-1)/mTOR pathway and promote the expression of genes involved in muscle protein synthesis (45, 65, 66). Similarly, Escherichia coli and Klebsiella play a role in the skeletal muscle anabolism and cell proliferation through the stimulation of the IGF-1/mTOR pathway (67).

Recently, Picca et al. profiled GM in older adults affected by physical frailty and sarcopenia, showing that an increase of Oscillospira and Ruminococcus, and a decreased of Barnesiellaceae and Christensenellaceae microbial taxa, are associated with muscle impairment (68). Furthermore, it has been reported that exercise induces changes of GM with an important over-representation of some healthy bacterial species, such as Akkermansia, Prevotella, Faecalibacterium, and Roseburia, in fecal samples of humans with active lifestyle (69–73).

Regardless the correlation between GM and muscle function, Bjørkhaugh et al. showed that patients with chronic alcohol overconsumption had a loss of muscle strength, assessed by hand-grip strength test, associated with a higher relative abundance of Proteobacteria, Sutterella, Clostridium and Holdemania and a lower relative abundance of Faecalibacterium with reduced SCFAs fecal levels (74). In a recent non-randomized trial examining the effect of exercise on GM modulation in 32 sedentary elderly women it has been reported that the abundance of Bacteroides was positively correlated with an increased physical performance assessed by the 6-min walking distance test (75). Furthermore Fielding et al. showed that the colonization of germ-free mice with GM of high-functioning older adults is associated with an increase of muscle strength with a higher relative abundance of Prevotella and Barnesiella (76).

Therefore, the modulation of GM modulation could impact significantly on the onset of sarcopenia. Varian et al. have shown that the administration of Lactobacillus reuteri in mouse models of cancer could inhibit the development of sarcopenia and increase in muscle weight and fiber size, through an up-regulation of the transcriptional factor Forkhead Box N1 (FoxN1) (77).

In a randomized controlled study prebiotic administration compared with placebo significantly improved two frailty criteria: exhaustion and increases the grip strength in elderly people over 65 years old (78). Similarly, Munukka et al. found that supplement of probiotics (Faecalibacterium prausnitzi) increased muscle mass that is linked to enhanced mitochondrial respiration, improved insulin sensitivity, modified gut microbiota composition and improved intestinal integrity (79).

Furthermore, folate and vitamin B12 are used as nutrient substrates by intestinal microflora for the maintenance of GM homeostasis; thus, nutrient deficiency negatively impacts on the GM function and consequently on muscle protein homeostasis (80).

### Malnutrition

The chronic inflammation contributes to malnutrition through associated anorexia and decreased food intake; it further impacts on metabolism with elevation of resting energy expenditure and increased muscle catabolism (81). Of note, in more than two thirds of malnourished patients, sarcopenia coexists (82).

Malnutrition is also a condition that commonly affect IBD patients. In detail it has been reported in 65–75% of patients with CD and in 18–62% of patients with UC, as results of malabsorption, side effects of steroids, and increase in basal energy expenditure due to the inflammatory status (19). Importantly, malnutrition is typically associated to a significant alteration of body composition i.e. weight loss, reduction of skeletal muscle mass, bone loss, and expansion of visceral and “creeping” fat (83, 84).

The association of malnutrition and sarcopenia in IBD patients is controversial. Indeed, it is noteworthy that while malnutrition in IBD is characterized by weight loss during the acute phase of disease followed by a gradual recovery during disease remission, sarcopenia may be present even in IBD patients in remission and not only with low but also normal or elevated values of BMI (85–87).

However, the poor and/or inadequate oral intake is considered one of the most important determinants of IBD-related malnutrition, due to a voluntary food restrictions and symptoms such as nausea, abdominal pain, vomiting, and diarrhea occurred in case of IBD flare up (88). Importantly, macronutrient intake is usually preserved in almost all IBD patients, while micronutrient deficiency, such as iron, copper, selenium, magnesium, zinc, vitamins and antioxidants, can occur more frequently (89). In addition, an insufficient protein intake can determine sarcopenia.

It is now clear that nutrients contribute significantly for the health of the trillions of bacteria, fungi, viruses, phages, parasites, and archea that compose the GM. Metagenomics analysis revealed that diet alters microbial community structure and overwhelms inter-individual differences in microbial gene expression (90–92). For instance, high-protein diets are associated with low microbial diversity. While a high-fat diet leads to a decrease in Bacteroidetes and an increase in Firmicutes, alterations that have been associated with an increase of opportunistic bacteria, intestinal permeability, low-grade systemic inflammation and insulin resistance (93). Conversely, promotion of insulin sensitivity, mitochondrial biogenesis, energy production and modulation of inflammation are induced by SCFAs produced by gut bacteria such as Faecalibacterium, Succinivibrio, and Butyricimonas (20). Of note, human studies focused on GM and malnutrition showed that children with protein energy wasting displayed an increase in Proteobacteria and a decrease in Bacteroidetes when compared with healthy children (94, 95).

Whilst there is a large body of supporting evidence for supplemental interventions for the prevention of muscle loss, strength and function in older adults, few data analyze this effect in patients with IBD.

Leucine supplementation, as well as vitamin D, in association with physical exercise increased skeletal muscle mass and muscle strength (96). Furthermore, the supplementation of beta-hydroxy-beta-methyl butyrate is associated with preservation of muscle tissue during short period of bed rest and increased muscle mass and strength, particularly in combination with resistance training (97–99).

Furthermore, vitamin D deficiency plays a key role in the onset of IBD-related sarcopenia (18). A recent metanalysis including 938 IBD patients, showed that lower vitamin D serum levels are more frequent in patients with IBD (64%) than controls (100). Although the exact mechanism through which vitamin D affects skeletal muscle homeostasis is not totally elucidated, loss of muscle mass seems to be strictly related to the decrease of vitamin D receptor (VDR) expression (101). It has been reported that vitamin D, once interacting with VDR, can elicit two different effects: 1) a non-genomic effect, such as modulation of calcium channel activation, muscle contraction and mitochondrial function; 2) a genomic effect, up-regulating nuclear expression of gene coding contractile proteins and myogenic transcription factors (102). It has been reported that VDR expression declines in elderly and in patients affected by chronic inflammatory diseases, such as IBD, chronic obstructive pulmonary disease, renal failure, diabetes and asthma (103, 104). Therefore, vitamin D serum levels should be monitored and supplemented to prevent the onset of osteoporosis and muscle wasting in IBD patients.

## Studies Exploring IBD and Sarcopenia

### Animal Studies

The exact pathogenesis of sarcopenia in IBD patients is still uncertain. Thus, in an attempt to elucidate the interaction between gut inflammation and muscle failure, experimental animal models were reproduced. These imply to collect gastrointestinal and muscle tissue samples, but also plasma and stools to assess serum markers of inflammation and perform microbiota analysis (21). The main mouse models of experimental IBD are induced by the intrarectal administration of trinitrobenzene sulphonic acid (TNBS), leading to a transmural colonic inflammation similar to that observed in CD patients, or through the oral intake of dextran sulphate sodium (DSS), promoting a histological intestinal inflammation similar to UC in humans (105).

It has been shown that skeletal muscle mass and proteins are reduced in murine models of TNBS-induced colitis, suggesting the linkage between IBD and muscle wasting (106). In this context, gut inflammation seems to be the trigger of skeletal muscle atrophy due to an accelerated rate of protein breakdown mediated by the up-regulation of the ubiquitin proteasome proteolytic pathway and enhanced expression of atrogin-1 and Murf-1 skeletal muscle atrophy-related genes (atrogenes) implicated in muscle protein breakdown (106).

Furthermore, a transcriptional upregulation of Murf-1, and consequent myofibril degradation was observed in mice affected by DSS-induced colitis. Indeed these latter differ from controls for the loss of skeletal muscle mass and decrease in muscle function, assessed by the significant reduction of the number of gastrocnemius myofibrils and the physical performance on rota-rod test (107). Similarly, a mild chronic gut inflammation caused by DSS accelerates the muscle dysfunction evaluated by using rotarod test, gait analysis, and grip strength test in α-synuclein mutant mice (108).

The analysis of molecular pathways of IBD-related sarcopenia assessed by using immunohistochemistry showed a down-expression of IGF1-R and Phospho-mTOR, markers of muscle growth, and an over-expression of Murf-1 and Myostatin, considered markers of sarcopenia (107). Similarly, Saul et al. showed that experimental IBD mice had a decrease in skeletal muscle weight and fiber size with a reduction of muscle protein content tested in quadriceps and gastrocnemius (109). Moreover, they observed an increased in mRNA expression of Murf‐1 and Atrogin-1 suggesting an enhanced protein degradation responsible of sarcopenia (109).

However human muscle stem cells (hMuSCs) treated with IFN-γ and TNF-α for 48 hours, and then transplanted intravenously, ameliorated colitis in mice treated with DSS by producing TNF-stimulated gene 6 (TSG-6) implicated in anti-inflammatory functions (110).

Notably, animal models have been used also to evaluate the effects of gut microbiota manipulation on parameters of muscle mass and function. Indeed, probiotics seem to have a marked anti-inflammatory effect with beneficial consequences for muscle health through the promotion of anabolism. Probiotics containing Faecalibacterium prausnitzii, one of the main SCFA producers, were associated with improved liver anabolism and reduced systemic inflammation in mice models (79).

### Human Studies

The assessment of nutritional status with body composition and an early detection of sarcopenia in patients with IBD is essential for providing an appropriate nutritional support, even during the remission period, and preventing sarcopenia-related surgical and negative outcome.

To date the majority of studies have defined sarcopenia as a loss of muscle mass. Typically, three imaging techniques have been employed for the assessment of muscle mass: computed tomography (CT), dual energy X-ray absorptiometry (DXA) and bioelectrical impedance analysis (BIA). While CT allows a direct estimate of muscle mass, DXA and BIA only provides indirect estimates such as lean mass. However, according to the revised European Working Group on Sarcopenia in Older People (EWGSOP), the definition of sarcopenia was also based on muscle function, quantity and physical performance (1). The muscle function is commonly evaluated through the handgrip strength, 5-times repeated chair stand test and 4-meter walking speed, while the physical performance can be measured by gait speed, the Short Physical Performance Battery, and the Timed-Up and Go test.

Ryan et al. performed a systematic review, including a total of 658 patients, to assess the prevalence of sarcopenia in IBD patients and the correlation between sarcopenia and needs of surgery and surgical outcomes in patients with IBD (4). Forty-two percent IBD patients had a diagnosis of sarcopenia detected with radiologic assessment of body mass composition and had an increased risk of requiring surgery with high rate of major complications after surgery (4).

Notably, sarcopenic IBD patients had lower preoperative serum levels of albumin and higher preoperative serum levels of C-reactive protein, deemed markers of malnutrition/inflammation and predictive factor of surgical negative outcome (86). Furthermore, a recent cross-sectional study involving 344 IBD patients in clinical remission revealed an increased risk of sarcopenia in malnourished patients (87).

However, in addition to muscle wasting, two thirds of IBD population had a lower perception of muscle strength with an increased asthenia and a decreased quality of life, similarly to geriatric controls; importantly, the combination of the quantitative and the qualitative parameters of muscular disorder configured a condition of sarcopenia in 28% of IBD patients (19).

Recently, mesenteric fat proliferation is gaining growing attention. Indeed, many studies support the active role of mesenteric fat creeping in the pathophysiology and clinical course of CD. Grillot et al. described the association between sarcopenia and visceral obesity assessed by computed tomography (CT) with adverse outcomes in severe CD patients, supporting the hypothesis that the human fat is considered as a dynamic tissue involved in the immunity regulation and consequent inflammation response (111).

Interestingly, the visceral to subcutaneous adipose tissue area ratio has been considered as a biomarker of complications of CD, such as stricture and fistula (111).

Bamba et al. observed that the muscle volume and visceral adipose tissue volume (relative to subcutaneous adipose tissue volume) are associated with the long-term outcome of intestinal resection (112). In detail, it has been shown that male sex, CD, low psoas muscle index, and high visceral adipose tissue volume are associated with bowel surgery; therefore, moderate exercise and elemental diet might be useful for maintaining muscle mass and reduce visceral fat (112).

Biologic therapy, including anti-TNF alpha agents and newer anti-interleukin, anti-integrin, and JAK inhibitors agents used for treatment of IBD, might reduce sarcopenia, blocking the catabolic effects on skeletal muscle tissue (54). Nevertheless, there are only few studies investigating the potential role of biologics in the prevention of muscle wasting in IBD patients. Subramaniam et al., showed that in active CD the treatment with infliximab can reverse IBD-related sarcopenia, leading to a significant improvement in both skeletal muscle volume and maximal isokinetic strength after 6 months of therapy (113). These highlight the key role of NF-kB signaling pathway in development of muscle impairment (113). Similarly, it was observed an improvement of BMI and muscle parameters, described as fat free mass index, after 3 months of infliximab or adalimumab, suggesting the beneficial effect of the anti-TNF alpha therapy on the nutritional status and body composition of IBD patients (114) (**Table 1**).

**Table 1:** Principal human studies that explored the relationship between inflammatory bowel diseases and sarcopenia.

Authors, Year	Study Design	Study Population/Number of patients	Intervention/Groups	Outcomes	Key findings
Subramaniam et al. (113), 2015	Prospective Study	99 patients with CD	MRI volume of quadriceps femoris, maximal concentric quadriceps contractions strength, physical activity, and serum levels of IL6 were assessed at week 1 (pretreatment),week 16 (post-IFX induction) and week 25 (post-first IFX maintenance dose)	Gain of muscle volume and strength after anti-TNF alpha therapy	The anti-TNF agent infliximab reverses inflammatory sarcopenia in patients with CD
Adams et al. (86), 2017	Retrospective Study	90 IBD patients	IBD patients starting a new anti-TNF alpha therapy that had CT within 3 months of initiation	Hospitalization, need for surgery, or new biological medication	45% of IBD patients were sarcopenic; of these, 19.5% were overweight/obese. CRP was higher and albumin lower in sarcopenic subjects. Sarcopenia was the only significant predictor of need for surgery in overweight and obese patients
Pizzoferrato et al. (19), 2019	Prospective Study	127 IBD patients	Four cohorts of patientswere recruited:	Rate of sarcopenia in IBD patients	36% of patients with IBD showed a significant reduction in skeletal muscle mass associated with a lower perception of muscle strength with a higher incidence of asthenia and reduction in quality of life
			IBD patients healthy controls healthy elderly elderly with primary sarcopenia		
Ryan et al. (4), 2019	Systematic Review	658 IBD patients	Five studies	Needs of surgery and surgical outcomes	42% of IBD patients had sarcopenia. IBD patients had a higher probability of requiring surgery. The rate of major complications was significantly higher in patients with sarcopenia
Grillot et al. (111), 2020	Retrospective Study	88 CD patients	CD patients who had abdominal CT scans during hospitalization	Prevalence of sarcopenia and visceral obesity in CD patients and its association with adverse events	The prevalence of sarcopenia was 58%. Among sarcopenic patients,13.7% were overweight, and 2% were obese. Sarcopenic CD patients had significantly more abscesses, hospitalizations and surgery. Both sarcopenia and visceral obesity were associated with adverse outcomes
Bamba et al. (112), 2020	Prospective Study	187 IBD patients	IBD patients who were admitted to hospital and underwent abdominal CT	Association of skeletal muscles and adipose tissue measured at the third lumbar vertebra level on CT image with clinical outcomes in IBD patients	Male sex, CD, low psoas muscle index, and high visceral to subcutaneous adipose tissue area ratio were associated with intestinal surgery
Ünal et al. (87), 2021	Cross-sectional Study	344 patients with IBD	IBD patients in clinical remission	Nutritional status and sarcopenia in patients with IBD in clinical remission	Sarcopenia was diagnosed in 41.3% of patients. 5.5% of patients were underweight and 9.9% were malnourished. Total number of flares requiring hospitalization was the most important predictor of sarcopenia

With regards to the effects of gut microbiota interventions on parameters of muscle mass and function, most of the available studies were carried out on animal models as we mentioned before. Few data have explored the impact of manipulating gut microbiota on skeletal muscle outcomes in humans. A randomized controlled trial (RCT) explored the effects of a mixture of inulin and fructooligosaccharides (FOS) versus placebo in ambulatory elderly residing in nursing homes for 13 weeks. Of note, the intervention group experienced significant improvements in handgrip strength and self-reported feeling of exhaustion (78). Subsequently Theou et al. conducted secondary analysis by using a similar intervention (inulin+FOS versus placebo) and showed improvements in physical function, frailty degree, nutritional status and quality of life following a 12-week intervention in frail elderly (115).

Thus, these data support the hypothesis of a modulation of muscle function by gut microbiota. However, translation of these results in IBD patients is uncertain. A clinical application of the basic science background linking gut microbiota with sarcopenia should be the challenge of future innovative research in IBD population. Hence further studies are awaited.

## Conclusions

The gut-muscle axis is a very promising area of research for the management of sarcopenia in IBD. It could be hypothesized that GM targeted treatment or complementary therapy such as physical activity and nutritional supplementation could be proposed to patients suffering from IBD and sarcopenia. Since sarcopenia has a negative impact on clinical outcome in IBD patients such as the need for surgical intervention and higher risk of post-operative complications, the assessment of muscle function and nutritional status should be adopted in the daily management of IBD patients. The modulation of the immune response, GM, oxidative stress, mitochondria disfunctions and metabolic processes could give potential benefits for IBD patients to improve muscle mass, muscle function and hence clinical outcomes. An incremental clinical benefit of blocking the catabolic effects on skeletal muscle tissue could be observed in IBD patients with sarcopenia treated by biologics including anti-TNF alpha agents and newer anti-interleukin, anti-integrin, and JAK inhibitors agents used for treatment of IBD. However, the evidence for GM targeting in IBD population is exiguous.

Understanding the pathophysiology of gut-muscle axis is key to developing translational research in this area of intense interest. Hence, we hope to encourage intervention studies exploring the impact of modulation GM on muscle function in IBD patients.

## Author Contributions

OMN and RdS contributed to conception, design, drafting, and revised of the manuscript. VP and FS wrote sections of the manuscript. AT and GV contributed to manuscript draft. FC critically reviewed the manuscript for important intellectual content, edited, revised, and provided overall supervision. All authors contributed to the article and approved the submitted version.

## Conflict of Interest

The authors declare that the research was conducted in the absence of any commercial or financial relationships that could be construed as a potential conflict of interest.
